# Redescription of *Japanagromyza inferna* Spencer, first recorded from Brazil, and a key to the Neotropical species of *Japanagromyza* Sasakawa (Diptera, Agromyzidae)

**DOI:** 10.3897/zookeys.374.6188

**Published:** 2014-01-28

**Authors:** Viviane Rodrigues de Sousa, Márcia Souto Couri

**Affiliations:** 1 Department of Entomology, Museu Nacional, Quinta da Boa Vista, São Cristóvão, Rio de Janeiro, 20940-040, Brazil

**Keywords:** Morphology, taxonomy, insect-plant interactions, gall-inducing, new records

## Abstract

*Japanagromyza inferna* Spencer is recorded for the first time from Brazil, in the North coast of the State of Rio de Janeiro, inducing galls in *Centrosema virginianum* L. (Fabaceae). The species is redescribed, with illustrations of male and female terminalia. A key to the identification of the Neotropical species of *Japanagromyza* Sasakawa is presented.

## Introduction

*Japanagromyza* Sasakawa has currently 80 known species in the world (Lonsdale 2013) and is represented by 30 in the neotropics (Martinez and Etienne 2002, Etienne and Martinez 2003, Sasakawa 2005, Boucher and Hanson 2006, Boucher 2010). Only one species has been recorded from Brazil (São Paulo), *Japanagromyza macroptilivora* Esposito & Prado (Esposito and Prado 1993). Some species are known to induce galls in plants, 15 of them are associated with plants of the Fabaceae family (Benavent-Corai et al. 2005) and other species are known to induce mines in crop plants (Spencer and Stegmaier 1973).

*Japanagromyza* is morphologically similar to *Agromyza* Fallén and *Melanagromyza* Hendel, although its species can be recognized by the following combination of characters: halter yellow, white, uniformly dark brown or variegated on top or inside of dark knob; thorax with two pairs of dorsocentral setae (rarely three pairs, but anterior ones only a little longer than acrostichals); one pair of scutellar setae (rarely absent); fore tibia with lateral setae in the middle (see Sasakawa 2010 for a complete description of the genus).

*Japanagromyza inferna* Spencer was originally described from Bahamas, with no information on the host plant (Spencer and Stegmaier 1973). Spencer et al. (1992) reported this species from Guadeloupe, also with no data on the host plant. Years later Etienne and Martinez (2003) recorded from Guadalupe and Saint Christopher, inducing leaf galls on *Centrosema virginianum* L. (Fabaceae). *Centrosema virginianum* is found throughout South America in forest scrub, “caatinga” and woodlands (Schultze-Kraft et al. 1990). Other species of Agromyzidae recorded as pests in plants of the genus *Centrosema* Benth. are *Ophiomyia centrosematis* (Meijere), *Melanagromyza phaseoli* Tryon, causing damage and influencing plant growth (Lenné et al. 1990), *Japanagromyza centrosematifolii* forming mines in *Centrosema virginianum* and *Centrosema pubescens* (Etienne and Martinez 2003) and *Japanagromyza centrosemae* Frost, known on *Centrosema pubescens* (Spencer 1990).

The main aim of this paper is to present a redescription of *Japanagromyza inferna*, including characters not yet described, and a key to the 30 Neotropical species of the genus *Japanagromyza*.

## Material and methods

**Figure 1.**
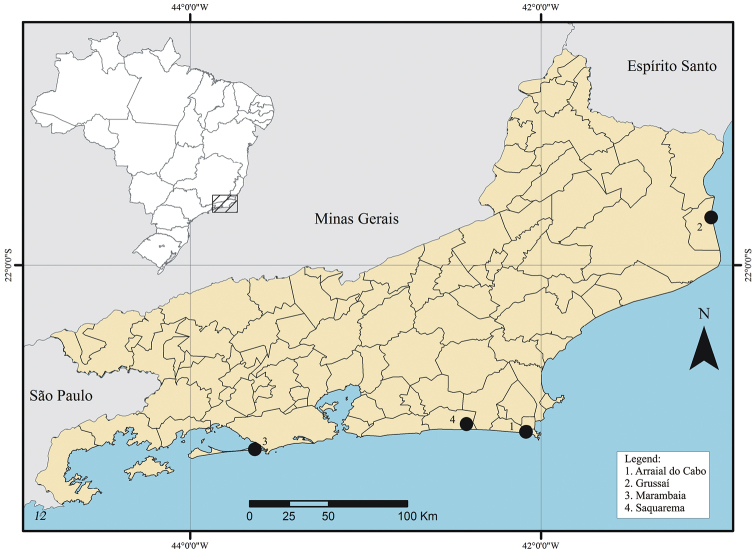
Map with the records localities of *Japanagromyza inferna* Spencer in Rio de Janeiro.

Collections were made bimonthly, from July 2011 to March 2012, in sandbanks in the North coast of the State of Rio de Janeiro (Fig. 1). The localities investigated were Arraial do Cabo, Grussaí (São João da Barra) and Saquarema (coordinates under material examined). In addition to these locations, an extra collection was made in the Marambaia sandbank, also located in Rio de Janeiro (Fig. 1).

To obtain material, branches of the plants with galls were removed and taken to the laboratory. The branches were placed in plastic pots, covered with organza and elastic for rearing and emergence of the adults. After emergence, adults were mounted on entomological pins and were deposited in the collection of Museu Nacional, Universidade Federal do Rio de Janeiro.

The terminalia were clarified in potassium 10% hydroxide for dissection under stereomicroscope and drawn using a camera lucida. Digital images of the gall, pupae and adult were prepared using a Leica MZ 16 optical microscope and the software program AutoMontage Pro by Syncroscopy. The species identification and the key to Neotropical species were based on the original descriptions. The terminology was based on Boucher 2010.

## Results

### Japanagromyza inferna

Spencer, 1973

http://species-id.net/wiki/Japanagromyza_inferna

#### Material examined.

BRAZIL, RIO DE JANEIRO: Saquarema: 22°56'06"S, 42°4'43"W. 3 ♂, 1 ♀, 01. VI. 2012. Col. V.R. Sousa; 22°56'03"S, 42°24'16"W. 3 ♂, 3 ♀, 18. XI. 2011. Col. V.R. Sousa; 1 ♂ and 2 ♀, 19 XI 2011. Col. V.R. Sousa. Arraial do Cabo: 22°57'00"S, 42°05'05"W. 1♀, 01. VI. 2012. Col. V.R Sousa. Grussaí: 21°43'42.5"S, 41°01'46.2"W. 1♂, 29. I. 2012. Col. V.R Sousa; 21°44'36.3"S, 41°01'44.7"W. 1♂, 01. II. 2012. Col. V.R. Sousa. Marambaia: 17 m 23°02'56"S, 43°37'51"W. 1 ♂, 4 ♀, 03. II. 2012. Col. V.R. Sousa. All forming galls in *Centrosema virginuanum* (L.) Benth.

#### Redescription.

**Figure 2.**
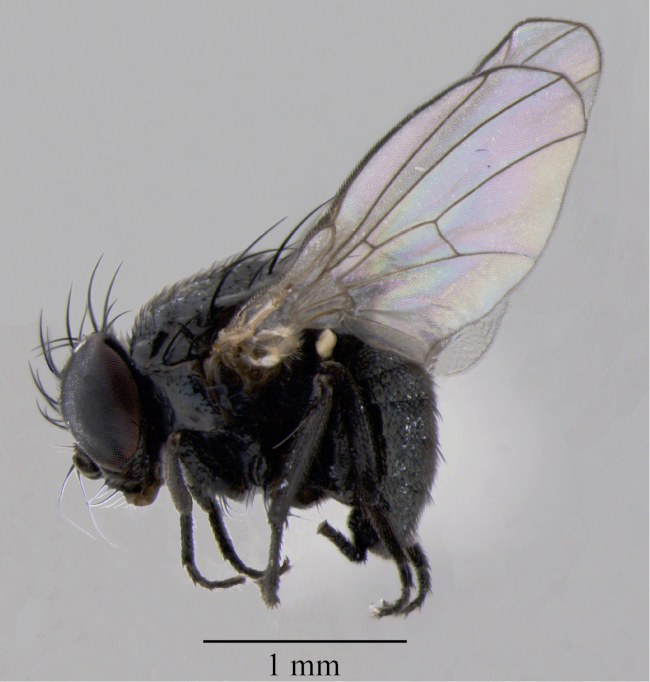
*Japanagromyza inferna* Spencer, male, in lateral view.

Male. (Fig. 2) - Body length: 2.5–2.6 mm. Wings length: 2.4 mm.

Color. Frons black dull, paler brownish at orbits level; face dark; fronto-orbital plate and ocellar triangle shining black; lunule gray pollinose; antenna black with apex of pedicel and base of postpedicel brown; arista black; palpus black; proboscis brown with labellum paler yellow with long yellow setae; thorax black with greenish reflections; halters yellow, brown at base; calypters and fringe yellow; legs black with coppery reflections; pulvilli white; abdomen black with coppery reflections.

Head. Fronto-orbital setulae in 4 pairs of rows, the two upper ors longer than the lower ones, first pair inclinate and the others posteriorly directed; ocellar triangle long; ocellar setae parallel and forward directed; internal orbital seta long, parallel and divergent; external orbital seta with about half the length of the internal; third antennal segment rounded and minutely pubescent; arista long and short pubescent; gena shorter with setae; vibrissa strong and short.

Thorax. Acrostichals in 10 rows, pre-sutural pair differentiated; two postsutural dorsocentral setae; two notopleural setae; one supra-alar; one intra-alar; one post-alar weak; two prospronotals; two pairs of scutellar setae, one sub basal and one apical, similar in size; four anepisternals with second upper one long and strong; katepisternum with small setae and one long katepisternal.

Legs. Fore tibia with one posterior supramedian seta. Mid tibia with two posterior setae inserted at middle third and one ventral apical seta. Hind tibia with one ventral apical seta.

**Figures 3–7.**
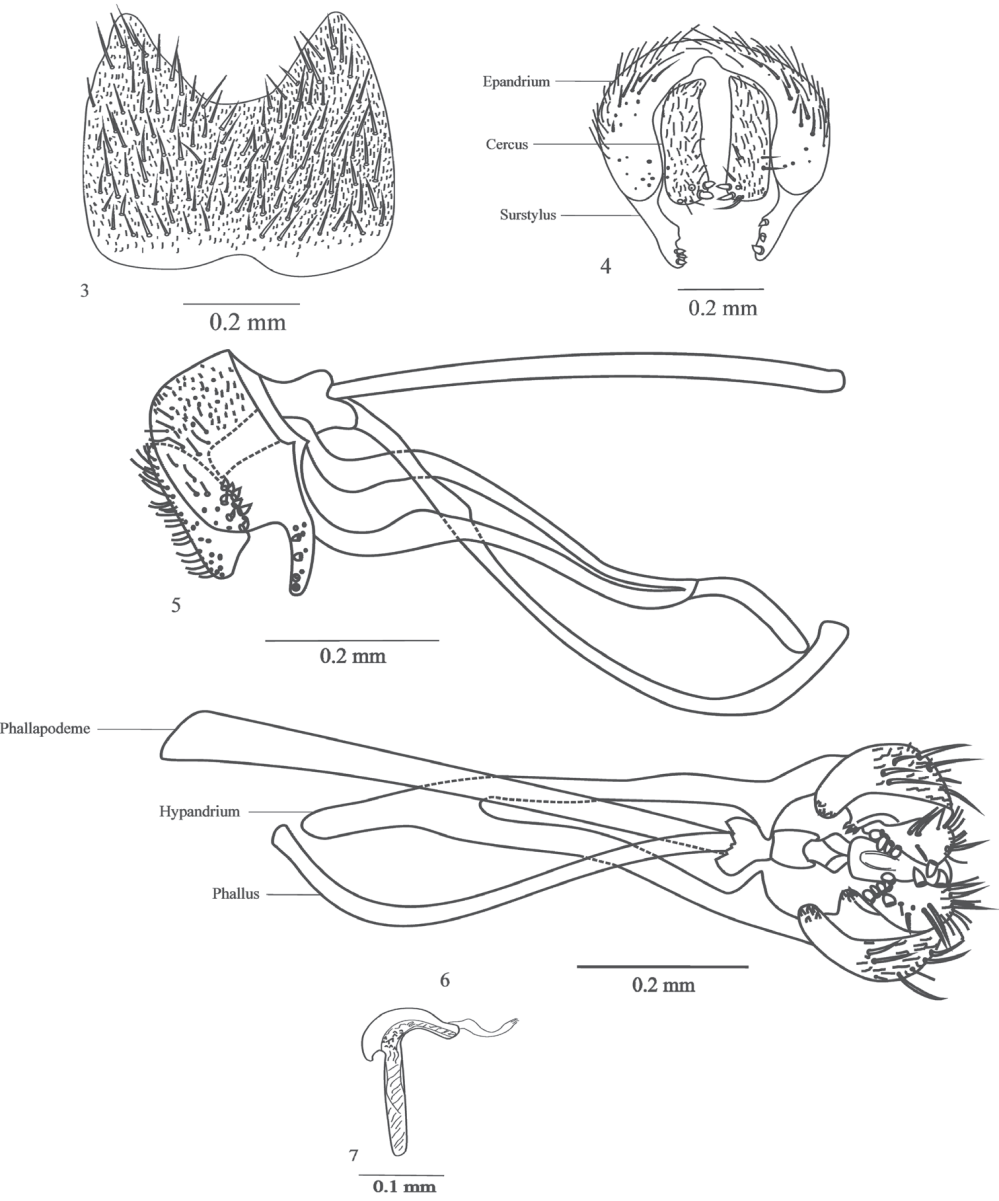
Male terminalia of *Japanagromyza inferna* Spencer **3** sternite 5 **4** epandrium, cercal plate and surstylus **5** hypandrium **6** phallapodeme, hypandrium, phallus **7** ejaculatory apodeme.

Abdomen. Sternite 5 large with setae in all its extension (Fig. 3).

Terminalia. Epandrium with internal margin with a small median indentation and with long setae; cercal plate with 3-4 spines in the basal portion, eight lateral spines and long cilia in all extension; surstylus long, slightly curved with about four thick spines (Fig. 4). Hypandrium v-shaped (Fig. 5); aedeagus simple, long and tubular, ornamented with membranes at the basiphallus (Fig. 6); ejaculatory apodeme small, hammer-shaped, with weak spines at base (Fig. 7).

Female. Similar to male.

**Figures 8–10.**
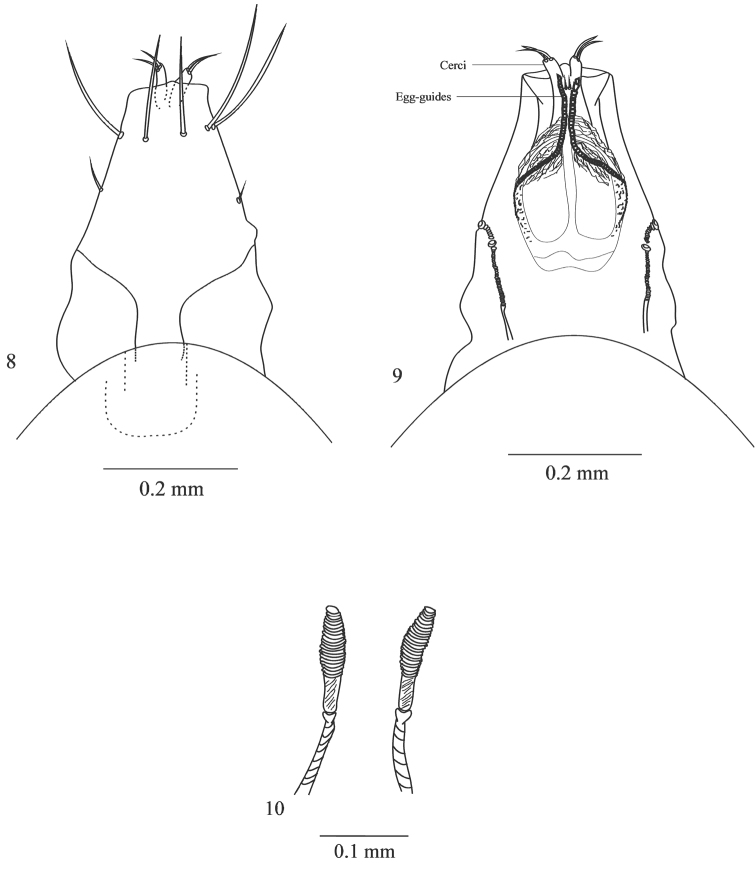
Female ovipositor of *Japanagromyza inferna* Spencer **8** dorsal view **9** ventral view **10** spermathecae.

Ovipositor. Dorsal view: cerci with two setae (Fig. 8). Ventral view: ninth tergite with one pair of long setae; egg-guides well sclerotized; two pairs of spiracles (Fig. 9). Spermathecae long and thin (Fig. 10).

**Figure 11.**
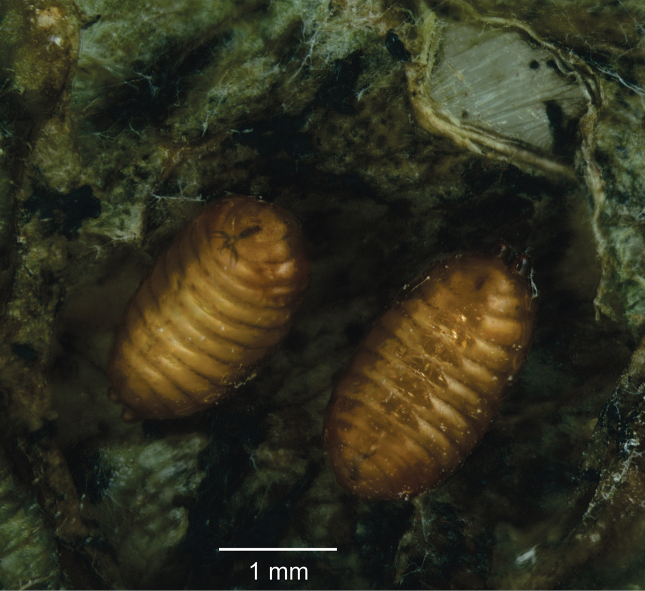
Pupae of *Japanagromyza inferna* Spencer in gall of the *Centrosema virginianum* L. (Fabaceae).

Puparium. General color orange-brown (Fig. 11).

#### Host-plant.

**Figure 12.**
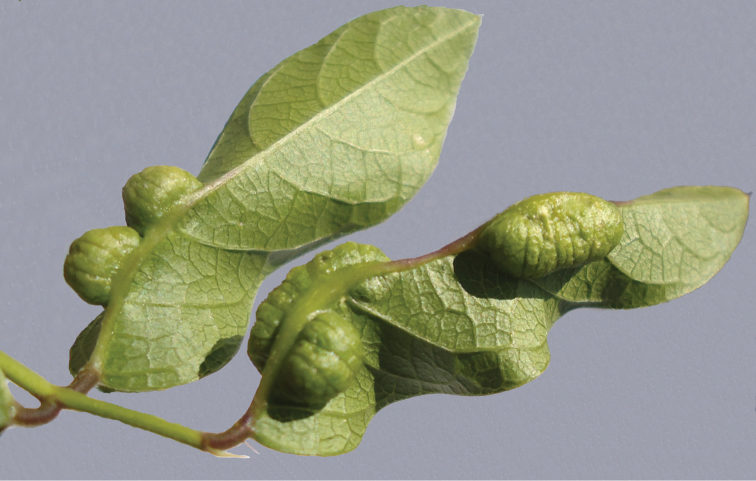
Gall of *Japanagromyza inferna* in *Centrosema virginianum* L. (Fabaceae).

*Centrosema virginianum*. Oval gall on leaf rib. 2–5 pupae on each gall. (Fig. 12).

#### Distribution.

Bahamas, Guadalupe, Brazil (Rio de Janeiro).

### Key to Neotropical species of *Japanagromyza*

**Table:** 

1	Pre-scutelar acrostichal setae absent	2
–	Pre-scutelar acrostichal setae present	5
2	Mesonotum distinctly greenish; two strong ors present; fringe of calypter white; male cerci without strong spines; shape of phallus as in figs 1–4 of Boucher and Hanson 2006 (Host-plant: *Lonchocarpus oliganthus*) [Costa Rica]	*Japanagromyza lonchocarpi* Boucher
–	Mesonotum greyish black; other combination of characters	3
3	Calypter dark grey, margin and fringe black (Host-plant: *Polygonum* sp.) [Venezuela, U.S.A (Florida)]	*Japanagromyza polygoni* Spencer
–	Calypter, margin and fringe whitish or silvery white	4
4	Abdomen greenish grey; arista bare (Host-plants: *Desmodium* sp., *Desmodium tortuosum*, *Desmodium campylocladus*) [Colombia, Equador, Peru, Venezuela, U.S.A. (Florida)]	*Japanagromyza desmodivora* Spencer
–	Abdomen shiny bluish black; arista plumose (Host-plant: unknown) [Peru]	*Japanagromyza tingomariensis* Sasakawa
5	Mesonotum distinctly greenish	6
–	Mesonotum greyish black	14
6	Halter with parts brown	7
–	Halter completely yellow or white	8
7	Frons gray dusted; lunule brown; acrostichals in 8 rows (Host-plant: unknown) [Colombia]	*Japanagromyza ambigua* Sasakawa
–	Frons black dull, paler brownish at orbits level; lunule gray pollinose; acrostichals in 10 rows (Host-plant: *Centrosema virginianum*) [Bahamas, Guadalupe, Brazil]	*Japanagromyza inferna* Spencer
8	Arista bare (Host-plant: *Macroptilium lathyroides*) [Brazil]	*Japanagromyza macroptilivora* Esposito & Prado
–	Arista distinctly pubescence or plumose	9
9	Frons uniformly brown (Host-plant: *Vigna luteola*) [Bahamas, Cuba, Guadalupe, La Dominica, U.S.A. (Florida)]	*Japanagromyza aequalis* Spencer
–	Frons black dull	10
10	Large species; wing length 3.1 mm; mesonotum uniformly greenish (Host-plant: unknown) [Panama, Porto Rico]	*Japanagromyza iridescens* (Frost)
–	Smaller species; wing length 1.9 to 2.4 mm; mesonotum greenish or coppery	11
11	Abdomen shiny greenish or faintly bluish; arista slightly pubescent (Host-plant: *Vigna luteola*) [Cuba, Barbade, Guadalupe, Dominica, Peru, Puerto Rico, Venezuela, Saint-Vicent, U.S.A. (Florida)]	*Japanagromyza inaequalis* (Malloch)
–	Abdomen strongly shining green; arista distinctly pubescent	12
12	Aedeagus consisting of a well-chitinized, flat basiphallus, an elongated membranous distiphallus (fig. 341, in Spencer and Stegmaier 1973); hypandrium V-shaped, with elongated hypandrial apodeme (fig. 342, in Spencer and Stegmaier 1973); surstyli extending downward, with about five short bristles on inner margin (fig. 343, in Spencer and Stegmaier 1973) (Host-plant: *Rhynchosia phaseoloides*) [Antigua, Barbados, Porto Rico]	*Japanagromyza bennetti* Spencer
–	Male terminalia other that described other above	13
13	Wing length 1.9 mm; aedeagus relatively short, as a membranous tubule; hypandrium with short, down-curved hypandrial apodeme (fig. 355 (A, B), in Spencer and Stegmaier 1973) (Host-plant: unknown) [Guadalupe, La Dominica]	*Japanagromyza wirthi* Spencer
–	Wing length 1.9 to 2.4 mm; aedeagus with basiphallus and median section uniformly but weakly chitinized, distiphallus entirely membranous; hypandrium rounded, without hypandrial apodeme (fig. 37 (A, B), in Spencer and Stegmaier 1973) (Host-plant: *Desmodium tortuosum*) [Bahamas, Costa Rica, Dominica, Guadalupe, Puerto Rico, Dominican Republic, Saint Martin, U.S.A. (Florida), El Salvador]	*Japanagromyza perpetua* Spencer
14	Halter entirely brown or black	15
–	Halter stem white or yellow, knob white or black	16
15	Fore tibia with distinct posterior setae; calypters largely brown or black (Host-plant: unknown) [Panama]	*Japanagromyza orbitalis* (Frost)
–	Fore tibia without setae; calypters yellow with margin and fringe pale brown (Host-plant: unknown) [El Salvador]	*Japanagromyza nebulifera* Sasakawa
16	Calypters gray with margin and fringe black; halter with knob black; (Host-plant: unknown) [Jamaica]	*Japanagromyza jamaicensis* Spencer
–	Calypters yellow with margin and fringe black or yellow; halter with knob yellow or black	17
17	Palpus yellow and abdomen with yellow areas	18
–	Palpus brown to black; abdomen normally shining black	19
18	Frons black dull behind, brownish in front; fringe of calypters yellow; aedeagus with distiphallus as a curve tubule with small, paired processes at end (Host-plant: unknown) [Cuba, Cayman Islands, Jamaica]	*Japanagromyza maculata* (Spencer)
–	Frons black dull, paler, more yellowish in front; fringe of calypter dark brown; aedeagus with distiphallus large, paired terminal processes (Host-plant: unknown) [Bahamas, Guyana, Jamaica]	*Japanagromyza spadix* (Spencer)
19	Knob of halter dark black	20
–	Knob of halter white or yellow	21
20	Arista conspicuously pubescent; mid tibiae with two posterodorsal setae (Host-plant: unknown) [Guatemala, Panama]	*Japanagromyza aldrichi* (Frick)
–	Arista almost bare or microscopically pubescent; mid tibiae with one posterodorsal setae (Host-plant: unknown) [Guatemala]	*Japanagromyza approximata* (Frost) (new comb. by Sasakawa 2005)
21	Fore and mid tibiae without distinct setae (Host-plant: *Centrosema pubencens*) [Panama]	*Japanagromyza centrosemae* (Frost)
–	Fore and mid tibiae with one or two setae differentiated	22
22	Fore tibia with one anterodorsal seta and mid tibia with two strong posterodorsal setae (Host-plants: *Centrosema virginianum*, *Centrosema pubescens*) [Guadalupe, Saint-Christopher]	*Japanagromyza centrosematifolii* Etienne
–	Fore and mid tibiae with setae present but different disposition on anterodorsal and posterodosal	23
23	Fringe of calypter silvery	24
–	Fringe of calypter white or yellow	26
24	Aedeagus with long straight distiphallus; cerci without setae (fig. 40, in Spencer and Stegmaier 1973) (Host-plants: *Castanea* sp., *Quercus rubra*, *Quercus* spp. [Puerto Rico, Gulf of Mexico, Canada, U.S.A. (Florida)]	*Japanagromyza viridula* (Coquillett)
–	Aedeagus with two coiled tubules; cerci large, with numerous strong setae	25
25	Length of the wing about 1.75 mm; spines on cercus and surstylus not numerous (fig. 2 in Martinez 1994) (Host-plants: *Phaseolus lunatus*, *Phaseolus* sp.) [Guadalupe, Jamaica, Saint-Christopher, Saint-Martin]	*Japanagromyza etiennei* Martinez
–	Length of the wing from 2.5-2.75 mm; spines on cercus and surstylus numerous (fig. 20 in Spencer 1983 (Host-plants: *Phaseolus* spp., *Phaseolus vulgaris*) [Argentina, Costa Rica, Peru, Venezuela]	*Japanagromyza phaseoli* Spencer
26	Antennae light brown, with postpedicel darkened distally (Host-plant: unknown) [El Salvador]	*Japanagromyza arnaudi* Sasakawa
–	Antennae entirely black	27
27	Arista bare; spines on cerci and aedeagus as in figs 9-10 of Spencer 1963 (Host-plant: unknown) [Costa Rica]	*Japanagromyza frosti* (Frick)
–	Arista pubescence or plumose; spines and aedeagus different from above	28
28	Mesonotum shining black, without reflections	29
–	Mesonotum black with coppery reflections (Host-plant: unknown) [Bahamas]	*Japanagromyza propinqua* Spencer
29	Abdomen black strongly shining; arista pubescent (Host-plant: unknown) [Colombia]	*Japanagromyza clausa* Sasakawa
–	Abdomen opaque dark brown; arista distinctly plumose (Host-plant: unknown) [Panama]	*Japanagromyza currani* (Frost)

## Supplementary Material

XML Treatment for Japanagromyza inferna
